# English Cocker Spaniels under primary veterinary care in the UK: disorder predispositions and protections

**DOI:** 10.1186/s40575-023-00136-x

**Published:** 2024-01-18

**Authors:** Karolina S. Engdahl, Dave C. Brodbelt, Carla Cameron, David B. Church, Dan G. O’Neill

**Affiliations:** 1 Department of Clinical Sciences, Swedish University of Agricultural Sciences, PO Box 7054, 750 07 Uppsala, Sweden; 2 Pathobiology and Population Sciences, The Royal Veterinary College, Hawkshead Lane, North Mymms, Hatfield, Herts AL9 7TA UK; 3 Clinical Science and Services, The Royal Veterinary College, Hawkshead Lane, North Mymms, Hatfield, Herts AL9 7TA UK

**Keywords:** VetCompass, Electronic patient record, EPR, Breed, Dog, Epidemiology, Primary-care, Veterinary, Pedigree, Purebred, English Cocker Spaniel

## Abstract

**Background:**

The English Cocker Spaniel (ECS) is one of the most popular dog breeds in the UK but information on disorder predisposition and protection is limited. Using anonymised veterinary clinical data from the VetCompass™ Programme, this study aimed to compare disorder predisposition and protection between the ECS and the remaining dogs under primary veterinary care in the UK during 2016. Electronic patient records for random samples of ECS and non-ECS were reviewed. The most common disorders diagnosed during 2016 were extracted and compared using multivariable logistic regression, controlling for confounders.

**Results:**

The analysis included random samples of 2510/10,313 (24.3%) ECS and 7813/326,552 (2.39%) non-ECS. After accounting for confounding by age, sex, bodyweight within breed-sex, insurance status and veterinary practice group, the ECS had increased odds of 21/43 (48.85%) disorders at fine-level precision, with highest odds for aural discharge (odds ratio (OR) 14.66, 95% confidence interval (CI): 7.73–30.90, *P* <  0.001) and keratoconjunctivitis sicca (OR 7.64, 95% CI: 4.33﻿–14.14, *P* <  0.001) and lowest odds for atopic dermatitis (OR 0.14, 95% CI: 0.05–0.31, *P* <  0.001) and allergy (OR 0.14, 95% CI: 0.06–0.28, *P* <  0.001).

**Conclusions:**

This study provides evidence for strong predisposition to aural and ocular disorders and protection from hypersensitivity disorders in the ECS. These results can aid dog owners, breeders, and veterinarians to better monitor health in ECS, and promote earlier diagnosis with improved prognosis. Further, the results can help breeding organisations establish key priorities the health-based reforms of the ECS.

## Plain English Summary

The English Cocker Spaniel is a popular dog breed in the UK but there is limited information regarding how healthy the breed is compared to dogs overall in the UK. The VetCompass™ Programme collects anonymised clinical notes on dogs attending first-opinion veterinary practices in the UK. Using VetCompass information, this study compared a range of common disorders in the English Cocker Spaniel and dogs that were not English Cocker Spaniel to identify which disorders had higher or lower risk in English Cocker Spaniels. The study builds on a previous publication exploring disease occurrence in the English Cocker Spaniel using data from the VetCompass™ Programme. Using information on 2510 English Cocker Spaniels and 7813 other types of dogs, the English Cocker Spaniel had higher risk of 21/43 (48.84%) and lower risk of 11/43 (25.58%) disorders compared to all remaining dogs. Disorders with the highest risk in English Cocker Spaniels included ear discharge (14.66 times the risk), keratoconjunctivitis sicca (dry eye, 7.64 times the risk), and musculoskeletal pain (7.06 times the risk), while disorders with the lowest risk included alopecia (hair loss), atopic dermatitis (skin disease associated with allergy), and allergy in general. This study shows that first-opinion clinical records can help us to better understand breed health. The results highlight ear and eye problems as key health issues for English Cocker Spaniels that owners and veterinarians should be especially vigilant about. This awareness can be also help breeding organisations and breeders to improve breeding decisions and through identification of key priorities for the health of the ECS.

## Background

Selective breeding during the domestication of the dog has resulted in 800 distinct breeds, many of which show exaggerated characteristics compared with their progenitor wild-type canines that were selected towards to enhance their usefulness and desirability to humans [1–3]. This selection for specific characteristics and behaviours has resulted in wide physical and behavioural diversity *between* breeds while paradoxically reducing physical, behavioural and genetic variation *within* breeds [4]. High levels of health problems within some breeds have been attributed to this loss of genetic heterogeneity and accumulation of detrimental genes as well as to exaggeration of anatomical features to levels of extreme conformation that can often combine to lead to suffering and reduced quality of life [4, 5]. However, reliable evidence on fuller health profiles that offer information on disorder predispositions and protections is limited for many breeds, even though this is critically needed to support effective progress towards reforming breed health [6].

The English Cocker Spaniel (ECS) was first recognised as a distinct breed by The Royal Kennel Club in 1893 and was originally selectively bred to suit the work and terrain for hunting woodcock [7]. The ECS is currently one of the most popular dog breeds within the UK, but is now owned mainly as a companion domestic pet rather than as a working animal. The ECS was the third most commonly registered breed with The Royal Kennel Club (UK) in 2022, the fourth most common breed under primary veterinary care in 2019 in the VetCompass™ Programme in the UK, and the fifth most commonly microchipped breed in the UK between 2004 and 2014 [3, 8, 9]. According to the breed standard, the ECS should be merry, gentle, affectionate, full of life and exuberant, with a silky coat and a strong and compact body, but this description offers little in terms of firm health information [7].

Disorder prevalence describes the proportion of affected individuals from some wider population [10]. In a previous VetCompass study on disorder occurrence in ECS, the disorders with the highest prevalence were periodontal disease (20.97%), otitis externa (10.09%), obesity (9.88%), anal sac impaction (8.07%) and diarrhoea (4.87%) [11]. In contrast to reporting the absolute number of disorders, disorder predisposition and protection offer an alternative perspective on breed health by reporting health as a value relative to the health of some other group of animals e.g. comparing disorder risk in one breed compared to all remaining dogs [12]. Predisposition describes an increased susceptibility to a certain disorder that could result from individual or combined risk factors such as genetics, conformation, sex, age, and environmental factors, while protection describes a reduced susceptibility [6]. The ECS has been reported in the literature as predisposed to several disorders including otitis externa [13–15], hypothyroidism [16], lipoma [17], mammary tumours [18, 19], periodontal disease [20], non-neoplastic anal sac disorders [21, 22], pancreatitis [22, 23] and keratoconjunctivitis sicca [22, 24, 25]. Protections reported in the ECS include osteoarthritis [22, 26], cruciate ligament disease [22, 27], elbow and hip dysplasia [22], and skin-related disorders such as chronic itching, dermatitis, allergic skin disorder, and alopecia [22]. Disorder predisposition offers nothing about the frequency of the disorder, so that a disorder with no breed predisposition may still carry high prevalence and therefore high overall health and welfare impact for a breed [10, 28]. Thus, access to information on both disorder prevalence and predisposition/protection is necessary for fuller understanding of the relevance of individual disorders to the overall health profile of a breed. Partially meeting this need, some previous studies have provided some useful information on disease occurrence and risk in the ECS but overall inference from these results is limited by differences introduced from studying different dog populations and using diverse data sources [14, 19, 22, 23, 27]. Further, the univariable analyses used in many of these early studies failed to account for major confounders such as age, sex, and bodyweight. To date, there is no published comprehensive summary of disorder predisposition and protection in the ECS across a wide range of disorders in a single dataset. Such information would assist kennel clubs to develop evidence-based breed-specific health plans to prioritise and address key health issues within breeds [29].

Population-based secondary data from primary-care veterinary practices offer many research benefits because of access to large datasets, the possibility to explore both common and rare events, diagnoses made by veterinary surgeons, contemporaneous recording of the clinical records at the time of the clinical events and good generalisability of the results to the wider dog population [30]. Our previous work has described common disorders diagnosed in ECS in the UK, which is useful to provide an evidence base on disorders that are leading contributors to the overall disorder burden in the breed [11]. However, this published descriptive information does not identify which disorders are more or less common in the ECS compared to other dogs in the UK. This comparative information is needed to identify further welfare opportunities for additional focus by breeders and owners. Using anonymised veterinary clinical data from the VetCompass™ Programme [31], this study aimed to evaluate disorder predisposition and protection between the ECS and the remaining non-ECS dogs under primary veterinary care in the UK during 2016. The specific objective was to report the odds ratios for the most common disorders in two groups of dogs: the ECS that were included in our previous study [11] compared to a group of non-ECS dogs, using multivariable modelling controlling for potential demographic confounding variables. Based on some prior evidence of predisposition to otitis externa [13–15], the study hypothesised that ECS have higher odds of ear disorders including otitis externa compared with non-ECS. These results could assist breeders, veterinary practitioners, and owners to detect, predict, prevent and manage key health and welfare opportunities for the ECS.

## Methods

The study population included all available dogs under primary veterinary care at clinics participating in the VetCompass™ Programme during 2016. Dogs under veterinary care were defined as those with either a) at least one electronic patient record (EPR) (free-text clinical note, treatment or bodyweight) recorded during 2016 or b) at least one EPR recorded during both 2015 and 2017. VetCompass collates de-identified EPR data from primary-care veterinary practices in the UK for epidemiological research [31]. Data fields available for the current study included a unique animal identifier along with species, breed, date of birth, sex, neuter status, insurance status along with bodyweight, treatment and free-form text clinical notes with relevant dates. Ethics approval was obtained from the RVC Ethics and Welfare Committee (reference number SR2018–1652).

A retrospective cohort study design was used that followed the clinical records of each dog in the analysis over a full year (2016) to benefit from the ability of this study design to evaluate multiple outcomes and account for several confounders in the analysis [32]. Contemporaneous recording of the clinical data at the time of each clinical event combined with blinding of the participating veterinary professionals to the research question being explored also reduced issues of recall and observer bias in the current cohort design compared to other observational study designs [10].

Breed information entered by the participating practices was cleaned and mapped to a VetCompass breed list derived and extended from the VeNom Coding breed list [33]. Dogs recorded as English Cocker Spaniel or Cocker Spaniel were categorised as ECS and all remaining dogs were categorised as non-ECS. Sample size calculations in *Epi info (CDC)* estimated that approximately 720 ECS and 3596 non-ECS were needed to detect an odds ratio of ≥ 1.5, based on an estimated 10% of ECS recorded with otitis externa during the study period as reported in our previous publication [11], with 80% power and 95% confidence assuming 5:1 ratio of non-ECS to ECS in the study population [34].

Neuter status was defined by the final available EPR neuter value and was combined with sex: female entire, female neutered, male entire and male neutered. Adult bodyweight was defined as the mean of all bodyweight (kg) values recorded for each dog after reaching 18 months old. Mean adult bodyweight was reported overall and broken down by sex for all breeds with adult bodyweight available for at least 100 dogs. Bodyweight was further categorised as “at or above the breed-sex mean”, “below the breed-sex mean” and “no recorded bodyweight”. Age (years) at the final study date (December 31, 2016) was categorised: ≤ 3.0, 3.0 to < 6.0, 6.0 to < 9.0, 9.0 to < 12.0 and ≥ 12.0. The data were derived from three veterinary practice groups (which were de-identified as A-C) including 510 clinics that were distributed across the UK. Insurance status was categorised as insured or not insured as recorded by the final available EPR.

The list of unique animal identification numbers for all dogs under veterinary care in 2016 was randomly ordered and the clinical records of randomly selected subsets of ECS and non-ECS were reviewed manually in detail to extract the most definitive diagnoses recorded for all disorders that existed during 2016 [11]. No information was extracted on the owner’s original rationale (e.g. seeking prophylactic care versus presenting a dog with prior awareness of health issues) for seeking veterinary care across the range of veterinary interactions that may have occurred during this year of interest. The extracted diagnosis terms were mapped to a dual hierarchy of diagnostic precision for analysis: fine-level precision and grouped-level precision as previously described [11, 35]. Elective (e.g. neutering) or prophylactic (e.g. vaccination) clinical events were not included. No distinction was made between pre-existing and incident disorder presentations.

Following data checking and cleaning in Excel (Microsoft Office Excel 2013, Microsoft Corp.), analyses were conducted using R version 4.2.1 [36]. The age, adult bodyweight, insurance status, and sex-neuter status for ECS and non-ECS under veterinary care during 2016 were described. Continuous variables were non-normally distributed and therefore summarised using median, interquartile range (IQR) and range. The Mann-Whitney U test and chi-square test were used as appropriate for comparison of demographic data between ECS and non-ECS [37, 38]. One-year period prevalence values were reported separately for ECS and non-ECS to describe the probability of diagnosis at least once during 2016. The final combined list of 43 fine-level disorders merged the 31 most common disorders in each of ECS and non-ECS, and the final combined list of 33 group-level disorders merged the 30 most common disorders in each of ECS and non-ECS. Multivariable modelling used binary logistic regression to report the odds for each disorder in these combined lists in ECS compared with non-ECS. A separate model was created for each fine-level and group-level disorder. Information theory was applied to generate a list of confounding variables that were consistently included alongside the breed variable in each model [39, 40]. Breed was an a priori factor of interest and the models additionally included age (years), at/above or below mean bodyweight for the breed-sex, insurance status, sex-neuter status, and veterinary practice group. Model fit was assessed with the Hosmer-Lemeshow Test [41]. Statistical significance was set at the 5% level.

## Results

### Demographics

The study population included 336,865 dogs under veterinary care during 2016, of which 10,313 (3.06%) were ECS and 326,552 (96.94%) were non-ECS. Random samples of 2510/10,313 (24.3%) ECS and 7813/326,552 (2.39%) non-ECS were included in the analysis, with descriptive results reported for these sampled dogs (Table 1). Median age did not differ between ECS (4.35 years, IQR 2.16–7.96, range 0.18–17.51) and non-ECS (4.42 years, IQR 2.17–8.05, range 0.22–20.46) (Mann-Whitney U test, *P* = 0.377). Median adult bodyweight of ECS (14.80 kg, IQR 12.90–17.00, range 8.40–23.20) was heavier than non-ECS (13.63 kg, IQR 8.00–25.10, range 1.41–85.00) (Mann-Whitney U test, *P* = 0.001). Data completeness were age 97.82%, bodyweight 66.78%, breed 99.49%, insurance status 100%, and neuter status 99.71%.

**Table 1:** Demographics in English Cocker Spaniels (ECS, *n* = 2510) and dogs other than ECS (non-ECS, *n* = 7813) under primary veterinary care in the UK during 2016. The *P*-value describes univariable comparison between ECS and non-ECS for that variable. ﻿*P*-values ≤ 0.05 are in bold

Variable	Category	ECS count (%)	Non-ECS count (%)	*P-*value
Age (years)	< 3	860 (34.36)	2670 (35.15)	**0.005**
	3 to < 6	712 (28.45)	2069 (27.24)	
	6 to < 9	447 (17.86)	1344 (17.70)	
	9 to < 12	337 (13.46)	914 (12.03)	
	≥ 12	147 (5.87)	598 (7.87)	
Sex and neuter status	Female entire	622 (24.79)	2210 (28.39)	**0.004**
	Female neutered	512 (20.41)	1548 (19.89)	
	Male entire	802 (31.96)	2294 (29.47)	
	Male neutered	573 (22.84)	1732 (22.25)	
At/above or below mean bodyweight for breed-sex	At or above	841 (33.51)	2320 (29.69)	0.394
	Below	950 (37.85)	2751 (35.21)	
	Not recorded	719 (28.65)	2742 (35.09)	
Insurance status	Not insured	2240 (89.24)	7212 (92.31)	**< 0.001**
	Insured	270 (10.76)	601 (7.69)	
Veterinary practice group	A	581 (23.15)	2025 (25.92)	**0.021**
	B	19 (0.76)	55 (0.70)	
	C	1910 (76.10)	5733 (73.38)	

### Disorder predispositions and protections

During 2016, there were 1769/2510 (70.48%) ECS and 5234/7813 (66.99%) non-ECS diagnosed with ≥ 1 disorder. After accounting for age, at/above or below breed-sex mean bodyweight, insurance status, sex-neuter status and veterinary practice group, ECS had 1.12 (95% CI 1.02–1.25, *P* = 0.023) times the odds of ≥ 1 diagnosis compared to non-ECS.

After accounting for confounding using multivariable methods, ECS had significantly increased odds of 21/43 (48.84%) fine-level disorders compared to non-ECS (Table 2). The disorders with the highest odds in ECS were aural discharge (OR 14.66 95% CI: 7.73–30.80; *P* <  0.001), keratoconjunctivitis sicca (OR 7.64; 95% CI: 4.33–14.14; *P* <  0.001), musculoskeletal pain (OR 7.06; 95% CI: 3.74–14.08; *P* <  0.001), subcutaneous mass (OR 4.92; 95% CI: 2.92–8.46; *P* <  0.001), and ear disorder (OR 3.59; 95% CI: 2.33–5.57; *P* <  0.001). English Cocker Spaniels had significantly reduced odds of 11/43 (25.58%) fine-level disorders, with the lowest odds being for alopecia (OR 0.35; 95% CI: 0.16–0.67; *P* = 0.003), atopic dermatitis (OR 0.14; 95% CI: 0.05–0.31; *P* <  0.001), and allergy (OR 0.14; 95% CI: 0.06–0.28; *P* <  0.001).

**Table 2:** Multivariable logistic regression odds ratios for a combined list of 43 from the 31 most commonly recorded fine-level disorders in each of English Cocker Spaniels (ECS) and non-ECS dogs under primary veterinary care at UK practices participating in the VetCompass™ Programme from 1 January to 31 December 2016. Regression modelling accounted for age, at/above or below breed-sex mean bodyweight, insurance status, sex-neuter status and veterinary practice group. * CI confidence interval. *P*-values ≤ 0.05 are in bold

Fine-level disorder	ECS count (%)	Non-ECS count (%)	Odds ratio (95% CI*)	*P*-value
Aural discharge	49 (1.95)	10 (0.13)	14.66 (7.73–30.80)	**< 0.001**
Keratoconjunctivitis sicca	40 (1.59)	16 (0.20)	7.64 (4.33–14.14)	**< 0.001**
Musculoskeletal pain	30 (1.20)	13 (0.17)	7.06 (3.74–14.08)	**< 0.001**
Subcutaneous mass	37 (1.47)	24 (0.31)	4.92 (2.92–8.46)	**< 0.001**
Ear disorder	45 (1.79)	40 (0.51)	3.59 (2.33–5.57)	**< 0.001**
Hair coat disorder	42 (1.67)	45 (0.58)	2.86 (1.87–4.38)	**< 0.001**
Post-operative complication	58 (2.31)	66 (0.84)	2.64 (1.84–3.78)	**< 0.001**
Mammary mass	35 (1.39)	44 (0.56)	2.60 (1.62–4.12)	**< 0.001**
Foreign body	64 (2.55)	73 (0.93)	2.60 (1.84–3.67)	**< 0.001**
Papilloma	32 (1.27)	43 (0.55)	2.27 (1.41–3.60)	**0.001**
Post-operative wound	66 (2.63)	95 (1.22)	2.13 (1.54–2.93)	**< 0.001**
Ocular discharge	32 (1.27)	47 (0.60)	2.03 (1.28–3.19)	**0.002**
Cataract	30 (1.20)	53 (0.68)	1.90 (1.18–3.00)	**0.007**
Periodontal disease	505 (20.12)	914 (11.70)	1.89 (1.67–2.14)	**< 0.001**
Anal sac impaction	195 (7.77)	331 (4.24)	1.82 (1.51–2.18)	**< 0.001**
Aggression	99 (3.94)	171 (2.19)	1.80 (1.39–2.32)	**< 0.001**
Dental disease	30 (1.20)	58 (0.74)	1.59 (1.01–2.47)	**0.041**
Conjunctivitis	74 (2.95)	148 (1.89)	1.49 (1.11–1.97)	**0.006**
Diarrhoea	119 (4.74)	259 (3.31)	1.43 (1.14–1.78)	**0.002**
Obesity	243 (9.68)	519 (6.64)	1.41 (1.20–1.66)	**< 0.001**
Otitis externa	261 (10.40)	580 (7.42)	1.40 (1.20–1.63)	**< 0.001**
Skin mass	77 (3.07)	172 (2.20)	1.38 (1.03–1.82)	**0.022**
Lameness	74 (2.95)	189 (2.42)	1.17 (0.88–1.53)	0.262
Lipoma	38 (1.51)	100 (1.28)	1.13 (0.76–1.65)	0.531
Undesirable behaviour	41 (1.63)	122 (1.56)	0.99 (0.69–1.41)	0.971
Heart murmur	41 (1.63)	128 (1.64)	0.99 (0.68–1.41)	0.951
Pyoderma	36 (1.43)	119 (1.52)	0.91 (0.62–1.31)	0.628
Vomiting	63 (2.51)	208 (2.66)	0.90 (0.67–1.19)	0.484
Flea infestation	44 (1.75)	161 (2.06)	0.85 (0.60–1.19)	0.362
Wound	40 (1.59)	144 (1.84)	0.82 (0.57–1.17)	0.288
Claw/nail injury	26 (1.04)	118 (1.51)	0.67 (0.42–1.01)	0.064
Kennel cough (infectious tracheobronchitis)	17 (0.68)	76 (0.97)	0.66 (0.38–1.10)	0.131
Pruritus	25 (1.00)	113 (1.45)	0.65 (0.41–0.98)	**0.050**
Skin cyst	15 (0.60)	79 (1.01)	0.55 (0.30–0.92)	**0.033**
Patellar luxation	14 (0.56)	82 (1.05)	0.48 (0.26–0.82)	**0.012**
Gastroenteritis	14 (0.56)	100 (1.28)	0.40 (0.22–0.67)	**0.001**
Overgrown nail(s)	61 (2.43)	475 (6.08)	0.38 (0.28–0.49)	**< 0.001**
Osteoarthritis	19 (0.76)	147 (1.88)	0.37 (0.22–0.59)	**< 0.001**
Retained deciduous tooth	9 (0.36)	77 (0.99)	0.35 (0.16–0.67)	**0.003**
Pododermatitis	14 (0.56)	114 (1.46)	0.35 (0.19–0.59)	**< 0.001**
Alopecia	9 (0.36)	76 (0.97)	0.35 (0.16–0.67)	**0.003**
Atopic dermatitis	5 (0.20)	103 (1.32)	0.14 (0.05–0.31)	**< 0.001**
Allergy	7 (0.28)	146 (1.87)	0.14 (0.06–0.28)	**< 0.001**

At a grouped level of disorder precision, after accounting for confounding using multivariable methods, ECS had significantly increased odds of 13/33 (39.39%) group-level disorders compared to non-ECS (Table 3). The group-level disorders with the highest odds in ECS were oral cavity disorder (not stated as related to dental disease, OR 5.04; 95% CI: 2.91–8.95, *P* <  0.001), foreign body (OR 2.60; 95% CI: 1.84–3.67, *P* <  0.001), complications associated with clinical care (OR 2.26; 95% CI: 1.76–2.89, *P* <  0.001), polyuria/polydipsia (OR 1.92, 95% CI: 1.12–3.25, *P* = 0.016) and intoxication (OR 1.86; 95% CI: 1.10–3.08, *P* = 0.017). English Cocker Spaniels had significantly reduced odds of 4/33 (12.12%) group-level disorders; skin disorders (OR 0.70, 95% CI: 0.60–0.80, *P* <  0.001), hernia (OR 0.52, 95% CI: 0.27–0.92, *P* = 0.036), incontinence (OR 0.44, 95% CI: 0.21–0.82, *P* = 0.017), and claw/nail disorders (OR 0.43; 95% CI: 0.34–0.54, *P* <  0.001).

**Table 3:** Multivariable logistic regression odds ratios for a combined list of 33 from the 30 most commonly recorded group-level disorders in each of English Cocker Spaniels (ECS) and non-ECS dogs under primary veterinary care at UK practices participating in the VetCompass™ Programme from 1 January to 31 December 2016. Regression modelling accounted for age, at/above or below breed-sex mean bodyweight, insurance status, sex-neuter status and veterinary practice group. * CI confidence interval. *P*-values ≤ 0.05 are in bold

Group-level disorder	ECS count (%)	Non-ECS count (%)	Odds ratio (95% CI*)	*P*-value
Oral cavity disorder (not stated as related to dental disease)	34 (1.35)	22 (0.28)	5.04 (2.91–8.95)	**< 0.001**
Foreign body	64 (2.55)	73 (0.93)	2.60 (1.84–3.67)	**< 0.001**
Complications associated with clinical care	116 (4.62)	159 (2.04)	2.26 (1.76–2.89)	**< 0.001**
Polyuria/polydipsia	23 (0.92)	37 (0.47)	1.92 (1.12–3.25)	**0.016**
Intoxication	24 (0.96)	41 (0.52)	1.86 (1.10–3.08)	**0.017**
Ophthalmological disorder	261 (10.40)	462 (5.91)	1.82 (1.54–2.13)	**< 0.001**
Dental disorder	539 (21.47)	1025 (13.12)	1.78 (1.58–2.01)	**< 0.001**
Ear disorder	350 (13.94)	645 (8.26)	1.75 (1.52–2.02)	**< 0.001**
Anal sac disorder	214 (8.53)	386 (4.94)	1.71 (1.44–2.04)	**< 0.001**
Mass	206 (8.21)	421 (5.39)	1.58 (1.32–1.89)	**< 0.001**
Male reproductive disorder	37 (1.47)	71 (0.91)	1.53 (1.01–2.29)	**0.042**
Obesity	243 (9.68)	519 (6.64)	1.41 (1.20–1.66)	**< 0.001**
Thin	42 (1.67)	101 (1.29)	1.33 (0.91–1.91)	0.128
Urinary tract disorder	37 (1.47)	86 (1.10)	1.31 (0.87–1.92)	0.179
Appetite disorder	22 (0.88)	54 (0.69)	1.29 (0.76–2.10)	0.323
Behavioural disorder	156 (6.22)	382 (4.89)	1.26 (1.03–1.52)	**0.020**
Female reproductive disorder	52 (2.07)	143 (1.83)	1.25 (0.89–1.73)	0.182
Heart disorder	59 (2.35)	169 (2.16)	1.09 (0.80–1.47)	0.584
Lethargy	29 (1.16)	85 (1.09)	1.04 (0.67–1.58)	0.844
Spinal cord disorder	23 (0.92)	72 (0.92)	1.00 (0.61–1.59)	0.991
Enteropathy	223 (8.88)	687 (8.79)	0.97 (0.83–1.14)	0.753
Musculoskeletal disorder	185 (7.37)	567 (7.26)	0.97 (0.81–1.16)	0.757
Neoplasia	117 (4.66)	358 (4.58)	0.97 (0.77–1.20)	0.755
Parasite infestation	86 (3.43)	284 (3.63)	0.94 (0.73–1.19)	0.598
Endocrine disorder	20 (0.80)	64 (0.82)	0.92 (0.54–1.50)	0.740
Respiratory tract disorder	84 (3.35)	279 (3.57)	0.92 (0.71–1.18)	0.525
Traumatic injury	86 (3.43)	287 (3.67)	0.90 (0.70–1.14)	0.385
Skin disorder	253 (10.08)	1038 (13.29)	0.70 (0.60–0.80)	**< 0.001**
Brain disorder	21 (0.84)	103 (1.32)	0.65 (0.39–1.02)	0.076
Collapsed	10 (0.40)	58 (0.74)	0.63 (0.30–1.19)	0.183
Hernia	12 (0.48)	71 (0.91)	0.52 (0.27–0.92)	**0.036**
Incontinence	10 (0.40)	68 (0.87)	0.44 (0.21–0.82)	**0.017**
Claw/nail disorder	90 (3.59)	601 (7.69)	0.43 (0.34–0.54)	**< 0.001**

## Discussion

This is the largest study to date reporting on disorder predisposition and protection specifically in the ECS. The study builds on the prevalence information reported in a preceding publication on demography and disorders in ECS by adding comparative risk values across a range of common disorders between ECS and the remaining dogs under primary veterinary care in the UK [11]. The hypothesis for predisposition to ear disorders including otitis externa was supported, with ECS having 1.75 the odds of ear disorders compared with non-ECS at group-level precision.

At a fine-level diagnostic precision, the ECS had higher odds of 21/43 (48.84%) disorders and reduced odds of 11/43 (25.58%) disorders compared to non-ECS. Thus, the ECS had significantly different odds to non-ECS for 32/43 (74.42%) fine-level disorders. Studies using a similar methodology have been published for several breeds, such as the Labrador Retriever (increased/decreased odds of 34.3%/20.0% disorders, respectively), Staffordshire Bull Terrier (increased/decreased odds of 11.1%/13.9% disorders), Pug (increased/decreased odds of 57.5%/17.5% disorders), English Bulldog (increased/decreased odds of 55.8%/14.0% disorders), and French Bulldog (increased/decreased odds of 46.5%/25.6% disorders) [12, 42–45].

These results suggest that the disorder profile of the ECS is quite different to the typical dog. Overall, ECS had 1.12 times the odds of having at least one fine-level disorder diagnosed compared to non-ECS, suggesting a somewhat higher disease burden in the ECS compared to non-ECS. However, an understanding of the fuller implications of this increased frequency risk on the overall disorder burden and welfare of ECS would require additional consideration of information on severity and duration across all these disorders that is largely not currently available [28].

### Ear disorders

In alignment with previous reports of predisposition to otitis externa in several studies [13–15], the current study also showed increased odds of several ear-related problems in the ECS, including aural discharge (OR 14.66), ear disorder (OR 3.59), and otitis externa (OR 1.40). Primary causes of otitis externa that can initiate the inflammatory process include allergic skin disease, endocrinopathy, keratinisation disorders, ectoparasites, foreign bodies, and immune-mediated disorders [46], of which allergic skin disease is reported as the most common [13, 14]. The current study does not suggest that allergic skin disease is a main trigger for otitis externa in the ECS since both the prevalence and the odds of atopic dermatitis (prevalence 0.20%, OR 0.14) and allergy (prevalence 0.28%, OR 0.14) were low. However, allergic skin disease could have been underdiagnosed if clinical signs other than otitis externa were mild, so further studies are needed to confirm the contribution of allergic skin disease to otitis externa pathogenesis in the ECS. Other primary causes, such as foreign body, might be important in the ECS; otitis externa caused by aural foreign bodies (grass awns) has been reported as more frequent in ECS than other breeds [13]. Further, the pendulous ear shape of the breed is likely an important predisposing factor to otitis externa, as this a widely acknowledged risk factor [13, 15, 47–49].

In addition to ear-related disorders, the ECS was also predisposed to hair coat disorders (OR 2.86). Both hair coat and ear disorders can be caused by vitamin A-responsive dermatosis, a cornification disorder that causes greasy hair coats and ceruminous otitis that has some evidence of predisposition in Cocker Spaniels although the condition is reportedly rare [50, 51]. A study of chronic, severe otitis reported distinctly different pathologic characteristics of the ear canal of Cocker Spaniels with most dogs of this breed showing a ceruminous tissue response pattern compared to other breeds where the most common pattern was fibrosis [52]. Ceruminous otitis in ECS could have contributed to the high odds of aural discharge in the current study (OR 14.66) but could also have been a clinical sign of hypothyroidism, a common trigger of otitis externa that debuts in middle-aged to older dogs and also affects the hair coat [46, 53]. In our earlier ECS study, the prevalence of ear disorders in the ECS was shown to increase with age [11], and increased odds of hypothyroidism have been reported in the ECS, although the prevalence was low (0.37%) [16].

### Ophthalmologic disorders

Predispositions to several eye disorders in ECS were identified in the current study, including keratoconjunctivitis sicca (OR 7.64), ocular discharge (OR 2.03), cataract (OR 1.90), and conjunctivitis (OR 1.49), as well as ophthalmological disorders at a grouped level (OR 1.82).

Cataract involves partial or total opacity of the lens or its capsule and is one of the most common causes of blindness in dogs [54]. Cases of primary cataract, inherited cataract, age-related cataract and cataracts due to progressive retinal atrophy have been reported in the ECS [55, 56]. Despite the predisposition reported in the current study, the prevalence of cataracts in the ECS in this study (1.20% compared to 0.68% in the non-ECS) was substantially lower than the 8.23% reported in a study using data from the Veterinary Medical Data Base of US referral caseloads from 1974 to 2003 [57], and the 7.8% reported in a population of Cocker Spaniels presented to an ophthalmologic clinic Brazil between 2005 and 2008 [58]. This difference could potentially be explained by referral bias, with more complicated cases or those likely to require complex surgical care being selectively biased towards in referral datasets [59]. In contrast, our study population included dogs examined at primary-care veterinary practices, where specialised ophthalmologic examinations might be performed less frequently.

The predisposition to keratoconjunctivitis sicca reported in ECS in the current study is supported by previous reports [24, 25]. An autoimmune aetiology accounts for the majority of keratoconjunctivitis sicca cases and the condition can be associated with other autoimmune disorders such as hypothyroidism, systemic lupus erythematosus, and atopy [60]. An immune-mediated multi-organ disease has been suggested to affect the ECS, causing keratoconjunctivitis sicca but also chronic pancreatitis, glomerulonephritis, anal sac disease, and xerostomia [61, 62]. The increased odds of ocular discharge and conjunctivitis also identified in the current study may be linked to keratoconjunctivitis sicca, as mucoid or mucopurulent ocular discharge is a common clinical sign of the condition [60]. In addition, keratoconjunctivitis sicca is known to be a common, underlying cause of conjunctivitis, but the diagnosis may be missed if a Schirmer tear test is not performed [60, 63], suggesting that the prevalence of keratoconjunctivitis sicca in the current primary care study is likely underestimated. This highlights the importance of performing a Schirmer tear test in ECS with conjunctivitis and/or ocular discharge of unknown cause.

### Musculoskeletal disorders

Previous research has reported breed protection in ECS from several orthopaedic disorders including osteoarthritis, hip dysplasia, cruciate ligament disease, elbow dysplasia, and stifle joint disease in general [22, 26, 27, 64–66]. In the current study, the ECS had significantly lower odds of osteoarthritis (OR 0.37) and patellar luxation (OR 0.48) compared to non-ECS, and the prevalence of both conditions was also low in ECS (0.76 and 0.56%, respectively). Further, no significant difference in the odds of lameness (OR 1.17, *P* = 0.262) or musculoskeletal disease in general (OR 0.97, *P* = 0.757) was found. These results contrast with findings for the Cavalier King Charles Spaniel, a Spaniel breed of similar size to the ECS but which has increased odds of patellar luxation and a substantially higher prevalence of both patellar luxation (3.3%) and osteoarthritis (2.6%) [67–69]. This indicates than factors other than just being of spaniel heritage affects the risk of orthopaedic disease. In a study based on Swedish insurance data, the ECS had a decreased risk of medial patellar luxation, but an increased risk of lateral patellar luxation compared to all other insured dogs [69].

In contrast, however, the ECS in the current study showed high odds for musculoskeletal pain (OR 7.06), which is a non-specific sign that could be associated with almost any condition affecting the musculoskeletal system. Immune-mediated polyarthritis is one such condition that the ECS is reportedly predisposed to along with several other immune-mediated disorders [62, 70–75]. Immune-mediated polyarthritis is associated with non-specific clinical signs such as stiffness, lameness, difficulties walking/standing, exercise intolerance, joint swelling, and pain, although the condition is poorly understood and diagnostically challenging [72, 76, 77]. Therefore, the condition could be underdiagnosed and might have contributed to some of the observed predisposition to musculoskeletal pain in the current study. Hypothyroidism has also been anecdotally linked to myopathies and neuropathy, which can be difficult to differentiate from myopathy, is known to affect dogs with hypothyroidism and could have also contributed to the observed predisposition to musculoskeletal pain [53]. Further, intervertebral disk degeneration is reported within the breed and potentially also contributed to the observed predisposition [78, 79].

### Oral cavity and dental disorders

Oral cavity disorders, excluding those stated in the clinical records to be directly related to dental disorders, had the highest odds at group-level precision (OR 5.04) although the prevalence was relatively low at 1.35% compared to 0.28% in the non-ECS. This disorder group included a long list of fine-level disorders such as difficulty with prehension of food, oral discharge, increased salivation, dysphagia, gingivostomatitis, oral pain, halitosis, tongue disorders, and abnormal salivary gland or mucocele. Several of these disorders could still have been truly linked to dental disease or the loose skin of the lips, despite not being clearly stated in the patient records. The odds of dental disorders overall were also increased at group-level precision (OR 1.78), as were the odds of periodontal disease at fine-level precision (OR 1.89). In addition to this latter predisposition, the prevalence of periodontal disease was high (20.12%), although even this value was likely underestimated as a thorough examination during general anaesthesia is needed to evaluate the full extent of periodontal disease [80]. Periodontal disease involves inflammation in the periodontium surrounding and supporting the teeth initiated by plaque build-up on the tooth surface, and dental disorders such as periodontal disease are reported to have the highest welfare impact of commonly occurring disorders in dogs [28, 80]. In our earlier study of disorder prevalence in the ECS, we highlighted the importance of thorough oral examination in ECS presenting at veterinary practices, followed by devising a prevention and treatment plan, and this recommendation is further supported by the results of the current study [11]. In addition, this emphasises the importance of tooth brushing in the ECS, which is reported as one of the most effective ways of removing plaque from the tooth surface [80]. Another oral cavity disorder previously reported in the ECS that potentially could increase the risk of periodontal disease is xerostomia, although this was not commonly diagnosed in the current study [62].

### Foreign bodies

Foreign body was the group-level disorder with the second highest OR (2.60), although this group included foreign bodies at any location in the body. Previous studies of oesophageal and gastrointestinal foreign bodies did not report the ECS as predisposed or overrepresented in the study populations [81–86]. However, Brant et al. (2021) reported increased odds of grass seed foreign bodies in Spaniels compared to Retrievers, and the ECS was the most common breed among the affected dogs in that UK based study [87]. Spaniels were also reported as predisposed to grass seed foreign bodies in an Australian study [88]. The busy and working nature of the breed has been suggested to explain the predisposition, due to a higher exposure to grass seeds [87, 88], although the pendulous ear carriage likely also contributes by locking in grass seeds, increasing the risk for migration down the ear canal [88]. Otitis externa is reported as the most common manifestation of grass seed foreign bodies, and, as previously mentioned, otitis caused by grass awns is more common in the ECS than in other breeds [13, 88]. This emphasises the importance of excluding foreign bodies in ECS presenting with ear-related clinical signs. Further, densely haired paws are reported to increase the risk of interdigital grass seed foreign bodies which might have contributed to the increased odds of foreign bodies in the current study, and owners of spaniels have been advised to keep their dog’s ears and paws trimmed [87, 89].

### Neoplastic disease

The odds of masses were increased (OR 1.58) in ECS at group-level precision, while no significant difference in the odds of neoplasia was identified compared to non-ECS (OR 0.97, *P* = 0.755). Previous studies have reported increased risk of malignant neoplasia and increased odds of tumours in general (OR 1.50) in Cocker Spaniels [90, 91]. At fine-level precision in the current study, increased odds in ECS were identified for subcutaneous (OR 4.92), skin (OR 1.38), and mammary (OR 2.60) masses, and papilloma (OR 2.27). Based on the available clinical data, it was not possible to further classify the subcutaneous and skin neoplasia but previous papers have reported predisposition to skin and subcutaneous tumours such as melanocytic tumours [91], lipoma [17], and histiocytoma [92] in the ECS. In addition, the ECS was among the top three breeds for incidence of sebaceous gland tumours in a study based on data from the Swiss Canine Cancer Registry and had increased odds of anal gland sac carcinoma in a study using data from three British pathology institutions [93, 94]. In contrast, a decreased risk has been reported for mast cell tumours, haemangioma/haemangiosarcoma, and osteoma/osteosarcoma in ECS [91].

A predisposition to mammary tumours in ECS is supported by previous studies [18, 19], while Egenvall et al. (2005) used Agria Pet Insurance data to report that 18% of ECS had developed mammary tumours by 10 years of age [95]. Increased odds of having multiple mammary tumours in ECS have also been reported [96]. The cause of such increased risk is unknown but the evidence for breed predisposition suggests a genetic influence on the tumour development [97]. Two human breast cancer genes, *BRCA1* and *BRCA2,* and the *CDK5RAP2* gene involved in the cell cycle regulation, have been linked with increased odds of mammary tumours in the English Springer Spaniel, a breed at high risk of mammary tumours which is closely related to the ECS [98, 99]. Further, mammary tumour-associated single nucleotide polymorphisms in the estrogen receptor 1 gene, a gene involved in reproductive function but also pathological processes such as breast cancer, have been found both in the English Springer Spaniel and in a group of other high-risk breeds, including the ECS [97]. Further studies on the aetiopathogenesis and risk of tumours in the ECS are warranted.

### Limitations

The current study had some limitations, including those previously reported for the use of primary care veterinary data in research [30, 100]. There was potential misclassification bias for the breed since both dogs listed as “Cocker Spaniel” and “English Cocker Spaniel” in the patient records were classified as ECS. Additionally, some of the non-ECS may have had partial ECS heritage. For example, the Cockapoo is a planned cross between the Cocker Spaniel and Poodle and is increasingly common in the UK [3]. All crossbreeds with ECS heritage were classified as non-ECS in the current study, but it could not be evaluated what proportion of non-ECS that were ECS crossbreeds and how these affected the results (i.e. to what extent these dogs resulted in underestimation of the breed effects). Despite the relatively large sample size of the current study, the power to detect different odds ratios between the ECS and non-ECS naturally decreases as the frequency of the disorders decreased, generating increasing potential for Type II error (false negative results). This highlights the importance of considering both the count, odds ratio, prevalence, and confidence intervals when interpreting the results. Predisposition and protection from disease is important, but the prevalence, severity, and duration of disease should also be considered when evaluating the overall impact of disease on breed health [28]. It is important to clarify that the current study reports the prevalence of diagnosis of disorders at primary-care veterinary practices in the UK and not the true prevalence of disorders that inherently exist in the ECS. It is also possible that there is a breed bias effect here, where veterinarians may diagnose disorders proportionately higher in breeds where there is prior awareness that these disorders are predisposed. Further, some disorders were syndromic in nature and may have been related to several underlying specific disorders. The disorders were recorded and classified based on the description in the EPRs. Thus, the accuracy of this classification depends on the completeness and correctness of the medical records, which might depend on factors such as veterinarians’ individual experiences, time availability, and the owners’ willingness to consent to further diagnostic work up. Taking this into consideration, there is a risk of misclassification of the disorders. Given that the current study applied multiple testing, there is a risk of some Type I error (false positive results). Individual results should be therefore considered exploratory rather than confirmatory, and interpretation on individual disorders should be considered in conjunction with previous evidence as well as also being subject to confirmation in future studies. Although the Royal College of Veterinary Surgeons (RCVS) does maintain a register of veterinary surgeons, there is no complete national register of veterinary clinics in the UK. The RCVS does maintain a register of veterinary practice premises accredited by the RCVS Practice Standards Scheme that included 3235 premises in 2020 but the true number of clinics is likely to be substantially higher [101]. The current study sample of 510 clinics therefore represents under 16% of all UK clinics so there is a possibility of selection bias for the clinic, veterinary professional and owner demographics included in the current analysis. Since the time of the work done on the current study, VetCompass has continued to accrue more practices to participate in this national welfare-focused research programme and currently has over 1800 UK participating clinics [31]. Consequently, newer studies that repeat the current work could mitigate some of these selection biases.

## Conclusion

The current study identified that ECS are predisposed to ear disorders including discharge and otitis externa, keratoconjunctivitis sicca, musculoskeletal pain, oral disorders, periodontal disease, and several neoplastic conditions such as subcutaneous, mammary, and cutaneous masses. Conversely, ECS are protected from several skin-related disorders such as allergy, atopic dermatitis, alopecia, and pododermatitis. These results can aid dog owners and veterinarians in monitoring the health of ECS, hopefully resulting in earlier diagnosis and a better prognosis. Further, the results can help breeding organisations and breeders to improve breeding decisions through the identification of key priorities for the health of the ECS.

## Data Availability

The dataset supporting the conclusions of this article will be made available in the RVC Research Online repository.

## Abbreviations

CI: confidence interval
ECS: English Cocker Spaniel
EPR: electronic patient record
IQR: interquartile range
non-ECS: not English Cocker Spaniel
OR: odds ratio

## References

[1] Jansson M, Laikre L (2014). Recent breeding history of dog breeds in Sweden: modest rates of inbreeding, extensive loss of genetic diversity and lack of correlation between inbreeding and health. J Anim Breed Genet..

[2] Wayne RK, Ostrander EA (2007). Lessons learned from the dog genome. Trends Genet..

[3] O'Neill DG, McMillan KM, Church DB, Brodbelt DC (2023). Dog breeds and conformations in the UK in 2019: VetCompass canine demography and some consequent welfare implications. PloS One..

[4] Hedhammar Å, Malm S, Bonnett B (2011). International and collaborative strategies to enhance genetic health in purebred dogs. Vet J..

[5] ICECDogs. International Collaborative on Extreme Conformations in Dogs. https://dogwellnet.com/icecdogs/. Accessed 19 September 2023.

[6] Gough A, Thomas A, O'Neill D (2018). Breed predispositions to disease in dogs and cats.

[7] The Kennel Club. Spaniel (Cocker). https://www.thekennelclub.org.uk/search/breeds-a-to-z/breeds/gundog/spaniel-cocker/. Accessed 27 October 2022.

[8] BBC NEWS. Dog map: Find the top pooch in your postcode. https://www.bbc.com/news/uk-england-27690167. Accessed 27 October 2022.

[9] UK Kennel Club. Breed registration statistics. https://www.thekennelclub.org.uk/media-centre/breed-registration-statistics/. Accessed 14 September 2023.

[10] Dohoo I, Martin W, Stryhn H (2009). Veterinary epidemiologic research.

[11] Engdahl KS, Brodbelt DC, Cameron C, Church DB, Hedhammar A, O'Neill DG (2023). Demography and disorders of English cocker spaniels under primary veterinary care in the UK. Canine Med Genet..

[12] Pegram C, Wonham K, Brodbelt DC, Church DB, O’Neill DG. Staffordshire bull terriers in the UK: their disorder predispositions and protections. Canine Med Genet. 2020;7(1):1–11.

[13] Saridomichelakis MN, Farmaki R, Leontides LS, Koutinas AF (2007). Aetiology of canine otitis externa: a retrospective study of 100 cases. Vet Dermatol..

[14] Zur G, Lifshitz B, Bdolah-Abram T (2011). The association between the signalment, common causes of canine otitis externa and pathogens. J Small Anim Pract..

[15] O'Neill DG, Volk AV, Soares T, Church DB, Brodbelt DC, Pegram C (2021). Frequency and predisposing factors for canine otitis externa in the UK - a primary veterinary care epidemiological view. Canine Med Genet..

[16] O'Neill DG, Khoo JSP, Brodbelt DC, Church DB, Pegram C, Geddes RF (2022). Frequency, breed predispositions and other demographic risk factors for diagnosis of hypothyroidism in dogs under primary veterinary care in the UK. Canine Med Genet..

[17] O'Neill DG, Corah CH, Church DB, Brodbelt DC, Rutherford L (2018). Lipoma in dogs under primary veterinary care in the UK: prevalence and breed associations. Canine Genet Epidemiol..

[18] Varney D, O'Neill D, O'Neill M, Church D, Stell A, Beck S, et al. Epidemiology of mammary tumours in bitches under veterinary care in the UK in 2016. Vet Rec. 2023;193(5):e3054. 10.1002/vetr.3054 37231594

[19] Rodriguez J, Santana A, Herraez P, Killick DR, de Los Monteros AE (2022). Epidemiology of canine mammary tumours on the canary archipelago in Spain. BMC Vet Res..

[20] O'Neill DG, Mitchell CE, Humphrey J, Church DB, Brodbelt DC, Pegram C (2021). Epidemiology of periodontal disease in dogs in the UK primary-care veterinary setting. J Small Anim Pract..

[21] O'Neill DG, Hendricks A, Phillips JA, Brodbelt DC, Church DB, Loeffler A (2021). Non-neoplastic anal sac disorders in UK dogs: epidemiology and management aspects of a research-neglected syndrome. Vet Rec..

[22] Wiles BM, Llewellyn-Zaidi AM, Evans KM, O'Neill DG, Lewis TW (2017). Large-scale survey to estimate the prevalence of disorders for 192 kennel Club registered breeds. Canine Genet Epidemiol..

[23] Watson PJ, Roulois AJ, Scase T, Johnston PE, Thompson H, Herrtage ME (2007). Prevalence and breed distribution of chronic pancreatitis at post-mortem examination in first-opinion dogs. J Small Anim Pract..

[24] Helper LC (1996). The tear film of the dog. Animal Eye Res..

[25] O'Neill DG, Brodbelt DC, Keddy A, Church DB, Sanchez RF (2021). Keratoconjunctivitis sicca in dogs under primary veterinary care in the UK: an epidemiological study. J Small Anim Pract..

[26] Anderson KL, O'Neill DG, Brodbelt DC, Church DB, Meeson RL, Sargan D (2018). Prevalence, duration and risk factors for appendicular osteoarthritis in a UK dog population under primary veterinary care. Sci Rep..

[27] Engdahl K, Emanuelson U, Höglund O, Bergström A, Hanson J (2021). The epidemiology of cruciate ligament rupture in an insured Swedish dog population. Sci Rep..

[28] Summers JF, O'Neill DG, Church D, Collins L, Sargan D, Brodbelt DC (2019). Health-related welfare prioritisation of canine disorders using electronic health records in primary care practice in the UK. BMC Vet Res..

[29] The UK Kennel Club. Breed health and conservation plans (BHCPs) https://www.thekennelclub.org.uk/health-and-dog-care/what-we-do-for-dog-health/breeding-resources-and-health-schemes/breed-health-and-conservation-plans/. Accessed 1 October 2023.

[30] O'Neill DG, Church DB, McGreevy PD, Thomson PC, Brodbelt DC (2014). Approaches to canine health surveillance. Canine Genet Epidemiol..

[31] VetCompass. VetCompass Programme. http://www.rvc.ac.uk/VetCOMPASS/. Accessed 5 January 2023.

[32] Euser AM, Zoccali C, Jager KJ, Dekker FW (2009). Cohort studies: prospective versus retrospective. Nephron Clin Pract..

[33] The VeNom Coding Group. VeNom Veterinary Nomenclature. http://venomcoding.org. Accessed 11 November 2023.

[34] Epi Info CDC. Centers for Disease Control and Prevention (US): Epi Info. https://www.cdc.gov/epiinfo/index.html. Accessed 9 November 2022.

[35] O'Neill DG, Church DB, McGreevy PD, Thomson PC, Brodbelt DC (2014). Prevalence of disorders recorded in dogs attending primary-care veterinary practices in England. PloS One..

[36] R Core Team (2021). R: a language and environment for statistical computing.

[37] Mann HB, Whitney DR (1947). On a test of whether one of two random variables is stochastically larger than the other. Ann Math Stat..

[38] Fisher RA (1922). On the mathematical foundations of theoretical statistics. Philos Trans A Math Phys Eng Sci..

[39] Piccininni M, Konigorski S, Rohmann JL, Kurth T (2020). Directed acyclic graphs and causal thinking in clinical risk prediction modeling. BMC Med Res Methodol..

[40] Vineis P, Illari P, Russo F (2017). Causality in cancer research: a journey through models in molecular epidemiology and their philosophical interpretation. Emerg Themes Epidemiol..

[41] Hosmer D, Lemeshow S (2000). Assessing the fit of the model.

[42] O'Neill DG, Sahota J, Brodbelt DC, Church DB, Packer RMA, Pegram C (2022). Health of pug dogs in the UK: disorder predispositions and protections. Canine Med Genet..

[43] Pegram C, Woolley C, Brodbelt DC, Church DB, O'Neill DG (2021). Disorder predispositions and protections of Labrador retrievers in the UK. Sci Rep..

[44] O'Neill DG, Skipper A, Packer RMA, Lacey C, Brodbelt DC, Church DB (2022). English bulldogs in the UK: a VetCompass study of their disorder predispositions and protections. Canine Med Genet..

[45] O'Neill DG, Packer RMA, Francis P, Church DB, Brodbelt DC, Pegram C (2021). French bulldogs differ to other dogs in the UK in propensity for many common disorders: a VetCompass study. Canine Med Genet..

[46] Paterson S (2016). Discovering the causes of otitis externa. In Pract..

[47] Perry LR, MacLennan B, Korven R, Rawlings TA (2017). Epidemiological study of dogs with otitis externa in cape Breton. Nova Scotia Can Vet J..

[48] Hayes HM, Pickle LW, Wilson GP (1987). Effects of ear type and weather on the hospital prevalence of canine otitis externa. Res Vet Sci..

[49] Cafarchia C, Gallo S, Capelli G, Otranto D (2005). Occurrence and population size of Malassezia spp. in the external ear canal of dogs and cats both healthy and with otitis. Mycopathologia..

[50] Mauldin EA (2013). Canine ichthyosis and related disorders of cornification. Vet Clin North Am Small Anim Pract..

[51] Hensel P (2010). Nutrition and skin diseases in veterinary medicine. Clin Dermatol..

[52] Angus JC, Lichtensteiger C, Campbell KL, Schaeffer DJ (2002). Breed variations in histopathologic features of chronic severe otitis externa in dogs: 80 cases (1995-2001). J Am Vet Med Assoc..

[53] Mooney CT (2011). Canine hypothyroidism: a review of aetiology and diagnosis. N Z Vet J..

[54] Engelhardt A, Stock KF, Hamann H, Brahm R, Grussendorf H, Rosenhagen CU (2008). A retrospective study on the prevalence of primary cataracts in two pedigrees from the German population of English cocker spaniels. Vet Ophthalmol..

[55] Donzel E, Arti L, Chahory S (2017). Epidemiology and clinical presentation of canine cataracts in France: a retrospective study of 404 cases. Vet Ophthalmol..

[56] Olesen HP, Jensen OA, Norn MS (1974). Congenital hereditary cataract in cocker spaniels. J Small Anim Pract..

[57] Gelatt KN, Mackay EO (2005). Prevalence of primary breed-related cataracts in the dog in North America. Vet Ophthalmol..

[58] Baumworcel N, Soares AM, Helms G, Rei PR, Castro MC (2009). Three hundred and three dogs with cataracts seen in Rio de Janeiro. Brazil Vet Ophthalmol..

[59] Bartlett PC, Van Buren JW, Neterer M, Zhou C (2010). Disease surveillance and referral bias in the veterinary medical database. Prev Vet Med..

[60] Kaswan RL, Salisbury MA (1990). A new perspective on canine keratoconjunctivitis sicca. Treatment with ophthalmic cyclosporine. Vet Clin North Am Small Anim Pract..

[61] Watson P (2012). Chronic pancreatitis in dogs. Top Companion Anim Med..

[62] Coddou MF (2020). A study of a multi-systemic immune mediated disease in the English cocker spaniel.

[63] Sanchez RF, Innocent G, Mould J, Billson FM (2007). Canine keratoconjunctivitis sicca: disease trends in a review of 229 cases. J Small Anim Pract..

[64] Taylor-Brown FE, Meeson RL, Brodbelt DC, Church DB, McGreevy PD, Thomson PC (2015). Epidemiology of cranial cruciate ligament disease diagnosis in dogs attending primary-care veterinary practices in England. Vet Surg..

[65] Engdahl K, Hanson J, Bergström A, Bonnett B, Höglund O, Emanuelson U. The epidemiology of stifle joint disease in an insured Swedish dog population. Vet Rec. 2021;189(3):e197. 10.1002/vetr.197 33645813

[66] Pegram C, Brodbelt DC, Diaz-Ordaz K, Chang Y, von Hekkel AF, Church DB (2023). Risk factors for unilateral cranial cruciate ligament rupture diagnosis and for clinical management in dogs under primary veterinary care in the UK. Vet J..

[67] O'Neill DG, Meeson RL, Sheridan A, Church DB, Brodbelt DC (2016). The epidemiology of patellar luxation in dogs attending primary-care veterinary practices in England. Canine Genet Epidemiol..

[68] Summers JF, O'Neill DG, Church DB, Thomson PC, McGreevy PD, Brodbelt DC (2015). Prevalence of disorders recorded in cavalier king Charles spaniels attending primary-care veterinary practices in England. Canine Genet Epidemiol..

[69] Engdahl K, Bergström A, Höglund O, Hanson J. The epidemiology of patellar luxation in an insured Swedish dog population. Prev Vet Med. 2023;220:106034. 10.1016/j.prevetmed.2023.106034 37801966

[70] Ravicini S, Kent A, Dunning M, Baines S, Clarke S, Allerton F (2023). Description and outcome of dogs with primary immune-mediated polyarthritis: 73 cases (2012-2017). J Small Anim Pract..

[71] Johnson KC, Mackin A. Canine immune-mediated polyarthritis. Part 1: pathophysiology. J Am Anim Hosp Assoc. 2012;48(1):12 10.5326/JAAHA-MS-5744 22190602

[72] Jacques D, Cauzinille L, Bouvy B, Dupre G (2002). A retrospective study of 40 dogs with polyarthritis. Vet Surg..

[73] Coddou MF, Constantino-Casas F, Scase T, Day MJ, Blacklaws B, Watson PJ (2020). Chronic inflammatory disease in the pancreas, kidney and salivary glands of English cocker spaniels and dogs of other breeds shows similar histological features to human IgG4-related disease. J Comp Pathol..

[74] Miller SA, Hohenhaus AE, Hale AS (2004). Case-control study of blood type, breed, sex, and bacteremia in dogs with immune-mediated hemolytic anemia. J Am Vet Med Assoc..

[75] Reimer ME, Troy GC, Warnick LD (1999). Immune-mediated hemolytic anemia: 70 cases (1988-1996). J Am Anim Hosp Assoc..

[76] Clements DN, Gear RNA, Tattersall J, Carmichael S, Bennett D (2004). Type I immune- mediated polyarthritis in dogs: 39 cases (1997-2002). J Am Vet Med Assoc..

[77] Stull JW, Evason M, Carr AP, Waldner C (2008). Canine immune- mediated polyarthritis: clinical and laboratory findings in 83 cases in western Canada (1991-2001). Can Vet J..

[78] Bergknut N, Egenvall A, Hagman R, Gustås P, Hazewinkel HAW, Meij BP (2012). Incidence of intervertebral disk degeneration-related diseases and associated mortality rates in dogs. J Am Vet Med Assoc..

[79] Cardy TJ, Tzounos CE, Volk HA, De Decker S (2016). Clinical characterization of thoracolumbar and lumbar intervertebral disk extrusions in English cocker spaniels. J Am Vet Med Assoc..

[80] Wallis C, Holcombe LJ (2020). A review of the frequency and impact of periodontal disease in dogs. J Small Anim Pract..

[81] Hayes G (2009). Gastrointestinal foreign bodies in dogs and cats: a retrospective study of 208 cases. J Small Anim Pract..

[82] Gianella P, Pfammatter NS, Burgener IA (2009). Oesophageal and gastric endoscopic foreign body removal: complications and follow-up of 102 dogs. J Small Anim Pract..

[83] Wyatt SR, Barron PM (2019). Complications following removal of oesophageal foreign bodies: a retrospective review of 349 cases. Aust Vet J..

[84] Bongard AB, Furrow E, Granick JL (2019). Retrospective evaluation of factors associated with degree of esophagitis, treatment, and outcomes in dogs presenting with esophageal foreign bodies (2004-2014): 114 cases. J Vet Emerg Crit Care (San Antonio)..

[85] Zann GJ, Jones SC, Selmic LS, Tinga S, Wanstrath AW, Howard J (2022). Long-term outcome of dogs treated by surgical debridement of proximal humeral osteochondrosis. Vet Surg..

[86] Brisson BA, Wainberg SH, Malek S, Reabel S, Defarges A, Sears WC (2018). Risk factors and prognostic indicators for surgical outcome of dogs with esophageal foreign body obstructions. J Am Vet Med Assoc..

[87] Brant BJ, Singleton DA, Noble PJM, Radford AD (2021). Seasonality and risk factors for grass seed foreign bodies in dogs. Prev Vet Med..

[88] Hicks A, Golland D, Heller J, Malik R, Combs M (2016). Epidemiological investigation of grass seed foreign body-related disease in dogs of the Riverina District of rural Australia. Aust Vet J..

[89] Johnston DE, Summers BA (1971). Osteomyelitis of the lumbar vertebrae in dogs caused by grass-seed foreign bodies. Aust Vet J..

[90] Bronden LB, Nielsen SS, Toft N, Kristensen AT (2010). Data from the Danish veterinary Cancer registry on the occurrence and distribution of neoplasms in dogs in Denmark. Vet Rec..

[91] Gruntzig K, Graf R, Boo G, Guscetti F, Hassig M, Axhausen KW (2016). Swiss canine Cancer registry 1955-2008: occurrence of the most common tumour diagnoses and influence of age, breed, body size, sex and neutering status on tumour development. J Comp Pathol..

[92] Rodriguez J, Santana A, Borzollino MA, Herraez P, Killick DR, de Los Monteros AE (2023). Epidemiology of canine cutaneous round cell tumours on the canary archipelago in Spain. Vet Comp Oncol..

[93] Graf R, Pospischil A, Guscetti F, Meier D, Welle M, Dettwiler M (2018). Cutaneous tumors in Swiss dogs: retrospective data from the Swiss canine cancer registry, 2008-2013. Vet Pathol..

[94] Polton GA, Mowat V, Lee HC, McKee KA, Scase TJ (2006). Breed, gender and neutering status of British dogs with anal sac gland carcinoma. Vet Comp Oncol..

[95] Egenvall A, Bonnett BN, Ohagen P, Olson P, Hedhammar Å, von Euler H (2005). Incidence of and survival after mammary tumors in a population of over 80,000 insured female dogs in Sweden from 1995 to 2002. Prev Vet Med..

[96] Edmunds G, Beck S, Kale KU, Spasic I, O'Neill D, Brodbelt D (2023). Associations between dog breed and clinical features of mammary epithelial neoplasia in bitches: an epidemiological study of submissions to a single diagnostic pathology Centre between 2008-2021. J Mammary Gland Biol Neoplasia..

[97] Borge KS, Melin M, Rivera P, Thoresen SI, Webster MT, von Euler H (2013). The ESR1 gene is associated with risk for canine mammary tumours. BMC Vet Res..

[98] Rivera P, Melin M, Biagi T, Fall T, Haggstrom J, Lindblad-Toh K (2009). Mammary tumor development in dogs is associated with BRCA1 and BRCA2. Cancer Res..

[99] Melin M, Rivera P, Arendt M, Elvers I, Muren E, Gustafson U (2016). Genome-wide analysis identifies germ-line risk factors associated with canine mammary tumours. PLoS Genet..

[100] O'Neill DG, James H, Brodbelt DC, Church DB, Pegram C (2021). Prevalence of commonly diagnosed disorders in UK dogs under primary veterinary care: results and applications. BMC Vet Res..

[101] Royal College of Veterinary Surgeons. In: RCVS facts 2020. RCVS; 2020. https://www.rcvs.org.uk/news-and-views/publications/rcvs-facts-2020/?destination=%2Fnews-and-views%2Fpublications%2F%3Ffilter-keyword%3D%26filter-type%3D18%26filter-month%3D%26filter-year%3D&&&type=rfst&set=true#cookie-widget. Accessed 13 Nov 2023.

